# FOXC1‐ATP7A Axis Activates PI3K/AKT Signaling to Suppress Cuproptosis and Drive Fibroblast Pathogenesis in Rheumatoid Arthritis

**DOI:** 10.1096/fj.202600492R

**Published:** 2026-04-15

**Authors:** Daomin Lu, Fang Tang, Zong Jiang, Wukai Ma, Changming Chen, Lei Hou, Qianwei Lu, Xueming Yao, Danyang Li, Weiya Lan

**Affiliations:** ^1^ Graduate School Guizhou University of Traditional Chinese Medicine Guiyang China; ^2^ Department of Rheumatology and Immunology The Second Affiliated Hospital of Guizhou University of Traditional Chinese Medicine Guiyang China; ^3^ Joint Orthopedics The Second Affiliated Hospital of Guizhou University of Traditional Chinese Medicine Guiyang China; ^4^ Department of Pharmacology, College of Pharmacy Harbin Medical University Harbin Heilongjiang China

**Keywords:** ATP7A, cuproptosis, FOXC1, PI3K/AKT signaling pathway, rheumatoid arthritis

## Abstract

Rheumatoid arthritis (RA) is a chronic autoimmune disease characterized by persistent synovial inflammation and progressive joint destruction. The underlying molecular mechanisms remain incompletely defined. Transcriptomic data of RA and normal synovial samples (GSE89408) were analyzed to identify the differentially expressed genes (DEGs). WGCNA, GO/KEGG enrichment, and PPI analysis were performed on the DEGs to screen for hub genes involved in RA progression. The proliferation, apoptosis, migration, and invasion of the MH7A fibroblast‐like synoviocytes were evaluated through routine functional assays. QRT‐PCR and Western blotting were conducted for molecular validation. Bioinformatics analyses identified FOXC1 as a hub transcription factor in RA and confirmed its co‐expression with ATP7A. FOXC1 was markedly upregulated in RA tissues and fibroblasts, where it enhanced ATP7A expression. Overexpression of FOXC1 or ATP7A promoted the proliferation, invasion, and migration of RA fibroblasts while inhibiting apoptosis. Knockdown of ATP7A abrogated the effects of FOXC1. Mechanistically, the FOXC1‐ATP7A axis activated PI3K/AKT/mTOR signaling and reduced markers of cuproptosis in the MH7A cells, suggesting an essential role in maintaining fibroblast pathogenicity. FOXC1 is a novel upstream regulator of ATP7A in synovial fibroblasts, and the FOXC1/ATP7A/PI3K/AKT pathway mediates RA pathogenesis by activating the synovial fibroblasts and suppressing cuproptosis. Targeting this axis may provide new therapeutic opportunities for RA.

## Graphical abstract

This graphical abstract illustrates the regulatory mechanism of the FOXC1‐ATP7A axis in Rheumatoid Arthritis (RA). Bioinformatics analysis identifies FOXC1 as a hub transcription factor that is significantly upregulated in RA synovial tissues and fibroblast‐like synoviocytes (FLSs). FOXC1 directly promotes the transcription of ATP7A, which in turn activates the downstream PI3K/AKT/mTOR signaling pathway. This activation leads to a significant suppression of cuproptosis (copper‐dependent cell death) and a reduction in apoptosis. Consequently, the FOXC1‐ATP7A‐PI3K cascade drives the aggressive phenotypes of FLSs, including enhanced proliferation, migration, and invasion, ultimately contributing to persistent synovial inflammation and joint destruction in RA.

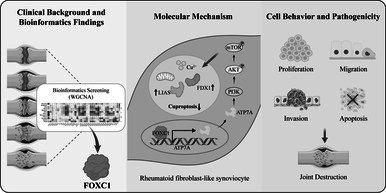

## 1 Introduction

Rheumatoid arthritis (RA) is a chronic autoimmune disease caused by a combination of genetic and environmental factors. The pathogenesis of RA is closely associated with the aberrant activation of diverse immune cell types in the synovium and peripheral blood, which trigger maladaptive tissue repair processes, eventually leading to synovial damage [1, 2, 3]. Synovial fibroblasts, also known as fibroblast‐like synoviocytes (FLS), maintain joint mobility by synthesizing extracellular matrix proteins and synovial fluid. However, during chronic inflammation, the FLS acquire a tumor‐like phenotype with increased proliferation, migration, and invasion, and play a major role in cartilage and bone destruction [4].

FOXC1, a key member of the Fox transcription factor family, regulates several biological processes such as metabolism, differentiation, proliferation, apoptosis, migration, invasion, and lifespan. Furthermore, FOXC1 promotes cell proliferation by upregulating the PI3K/AKT signaling pathway [5, 6]. Reports indicate significantly higher expression of FOXC1 in the FLS of patients with RA, where it co‐localizes with THY1, a specific marker of FLS, and promotes cellular proliferation via the PI3K/AKT signaling pathway [5].

ACPA, an autoantibody specific for cyclic citrullinated peptide (CCP), is an early diagnostic marker for RA and can also predict the risk of RA onset [7]. Through bioinformatics analysis, we found that ATPase copper transporting alpha (ATP7A) correlated significantly with the expression of ACPA. ATP7A and other copper transporting proteins pump excess copper ions out of cells and have been implicated in tumor invasion and metastasis [8]. Inhibiting the expression of ATP7A can disrupt copper transport mechanisms and homeostasis, leading to increased intracellular levels of copper ions, which suppress tumor development [9]. A Mendelian randomization analysis [10] reported differential expression of ATP7A in normal tissues and RA tissues, with significantly higher expression levels of ATP7A in RA tissues compared to non‐RA tissues [11]. However, its mechanism of action has not been reported so far. To this end, the aim of this study was to explore the interaction between ATP7A and FOXC1 in the pathogenesis of RA and investigate potential therapeutic approaches. We hypothesized that the FOXC1‐ATP7A axis drives RA pathogenesis by activating PI3K/AKT signaling and suppressing cuproptosis in the synovial fibroblasts.

## 2 Methods

### 2.1 Data Acquisition

Gene expression profile datasets were downloaded from the Gene Expression Omnibus (GEO) database (https://www.ncbi.nlm.nih.gov/geo). The GSE89408 dataset includes data of 28 healthy synovial samples and 152 RA samples. The platform and series matrix files were downloaded from GEO and saved in the TXT format. The probes were converted into corresponding gene symbols based on the relevant annotation information on the platform. For gene symbols with multiple probes, the average value was taken as the final expression value. The differentially expressed genes (DEGs) between the RA and control samples were screened.

### 2.2 Weighted Gene Co‐Expression Network Analysis (WGCNA)

The R package WGCNA [12] was used to identify the co‐expressed gene modules from the DEGs. The WGCNA network was established and the gene modules were detected using a soft‐threshold power *β* = 12. Genes in the core module were selected for further analysis. The module membership (MM), that is, correlation of each gene with its module eigengene, and the gene significance (GS)—the correlation between the gene and external traits—were quantified.

### 2.3 Protein–Protein Interaction (PPI) Network Analysis

The PPI network of the DEGs was constructed and visualized using the Cytoscape software [13]. The Molecular Complex Detection (MCODE [14]) plugin was used to extract the core modules in the PPI network, and the parameters were set as follows: degree cutoff = 2, K‐Core = 2, Node Score Cutoff = 0.2. The genes in the core module were regarded as potential hub genes.

### 2.4 Functional Annotation of DEGs

The target genes were functionally annotated using the “clusterProfiler” R package [15]. The gene ontology (GO) database was used to annotate the gene with terms related to biological pathways (BP), cellular components (CC), and molecular functions (MF). The pathways enriched in the DEGs were screened from the Kyoto Encyclopedia of Genes and Genomes (KEGG) database. The GO terms or KEGG pathways with adjusted *p* values < 0.05 were considered statistically significant.

### 2.5 Establishment of Murine Collagen‐Induced Arthritis (CIA) Model

Thirty male DBA/1 mice (8 weeks old) were purchased from SLC, Shizoka, Japan. The mice were divided into three groups—control, AAV‐shNC/CIA, and AAV‐shFOXC1/CIA. The model of CIA was established as described in the literature, and the severity of arthritis was assessed by evaluating clinical (arthritis score and paw thickness) and histological (synovial proliferation, bone destruction, and inflammatory cell infiltration through hematoxylin and eosin staining) indices. As per the experimental requirements, the mice were sacrificed and their spleens were extracted. T cells were isolated from the homogenized spleen tissues and analyzed using fluorescence‐activated cell sorting (FACS). All experimental protocols were approved by the Institutional Animal Care and Use Committee of Guizhou University of Traditional Chinese Medicine (License No. 20241107001).

### 2.6 Cell Culture

The in vitro experiments were conducted using the RA‐FLS line MH7A, which responds to TNF‐α and IL‐1β, and induces production of IL‐6 and other pro‐inflammatory factors. Briefly, primary synovial cells were isolated from the synovial tissue of a female RA patient who underwent total joint replacement, and immortalized through transfection with the SV40 large T antigen (SNL‐729, Wuhan Sunncell Biotechnology Co. Ltd.). The MH7A cells were cultured in DMEM supplemented with 10% fetal bovine serum and 1% penicillin–streptomycin at 37°C under 5% CO_2_. The cells were transfected with siRNA or overexpression plasmids using Lipofectamine 3000 (Life Technologies Inc. Carlsbad, California, USA), and harvested 48 h post‐transfection for subsequent experiments.

### 2.7 Quantitative RT‐PCR (qRT‐PCR)

Total RNA was isolated from MH7A cells using TRIzol reagent (Invitrogen, Carlsbad, CA, USA), and then reverse transcribed into cDNA using the First Strand cDNA Synthesis Kit (Thermo Fisher Scientific, Waltham, MA, USA) according to the manufacturers' instructions. RT‐qPCR was performed on the CFX96 Real‐Time RT‐PCR Detection System (Bio‐Rad, Hercules, CA, USA) with SYBR Premix Ex Taq Kit (TaKaRa Biotechnology, Tokyo, Japan) in a 25 μL reaction mixture for 40 cycles (95°C for 15 s, 62°C for 60 s, 72°C for 30 s). The Ct values of the samples were calculated, and transcript levels were analyzed using the 2^−ΔΔ*Ct*^ method.

### 2.8 Western Blotting

The cultured cells were washed with ice‐cold PBS and lysed radioimmunoprecipitation assay (RIPA) buffer containing protease and phosphatase inhibitors. The total protein concentration was measured using a BCA protein assay kit (Beyotime Biotechnology, Shanghai, China). Equal amounts of protein samples were diluted in loading buffer, denatured at 100°C for 10 min, and then separated by sodium dodecyl sulfate‐polyacrylamide gel electrophoresis (SDS‐PAGE) on a 10% gel. The protein bands were transferred to a polyvinylidene difluoride (PVDF) membrane (Millipore Corp, Billerica, MA, USA). After blocking with 5% skim milk in Tris‐buffered saline (TBS) containing 1% Tween‐20 at room temperature for 2 h, the membrane was incubated overnight with specific primary antibodies at 4°C. The membranes were then washed thrice with TBST, and incubated with horseradish peroxidase (HRP)‐conjugated secondary antibodies (1:10000 dilution) at room temperature for 1 h. Protein bands were detected using an enhanced chemiluminescence (ECL) detection system (Thermo Fisher Scientific Inc., Waltham, MA, USA) and quantified using ImageJ software.

### 2.9 Wound Healing Assay

MH7A cells transfected with either FOXC1 overexpression plasmid or the empty vector were seeded into cell culture plates and cultured for 24 h. The monolayer was scratched with a sterile pipette tip, and then washed with PBS to remove the dislodged cells and debris. Serum‐free medium was added, and the cells were cultured for 48 h. Images of the wound region were captured at 0 and 48 h, and the area was measured using ImageJ software. Cell migration rate was calculated based on the reduction in wound width.

### 2.10 Transwell Invasion Assay

The upper chambers of Transwell inserts were coated with Matrigel (Matrigel BD Biosciences, New York, USA) and inoculated with MH7A cells in 200 μL serum‐free medium. The inserts were placed in a 24‐well culture plate, and the lower chambers were filled with 800 μL DMEM containing 10% FBS. After 24 h of incubation, the cells were removed from the upper chamber, and the invasive cells were fixed and stained. All images were captured using a microscope (Olympus, Tokyo, Japan).

### 2.11 CCK8 Assay

The proliferation ability of MH7A was evaluated by the CCK‐8 assay (Dojindo, Osaka, Japan). MH7A cells were seeded in a 96‐well plate at the density of 5 × 10^3^ cells/well. At pre‐determined time points (24, 48, 72, and 96 h), 10 μL CCK‐8 reagent was added to each well, and the cells were incubated at 37°C for 1 h. The absorbance of each well at 450 nm was measured using a microplate reader (Bio‐Rad, California, USA), and cell proliferation was calculated based on the standard curve.

### 2.12 EdU Incorporation Assay

EdU incorporation assay was performed using the BeyoClick EdU‐555 Detection Kit (Beyotime, Shanghai, China). The transfected MH7A cells were seeded in 6‐well plates in complete medium and cultured for 24 h. The cells were then incubated with 50 mM EdU for 6 h, fixed, and stained for 30 min. Hoechst 33342 was used for nuclear staining. All images were captured using a fluorescence microscope.

### 2.13 Statistical Analysis

The data are presented as mean ± SEM. Multiple groups were compared by one‐way analysis of variance (ANOVA), and Student's *t*‐test was used for pairwise comparisons. Statistical analysis was conducted using GraphPad Prism 9.0 software. *p*‐values < 0.05 were considered statistically significant.

## 3 Results

### 3.1 Screening of Gene Co‐Expression Modules

**FIGURE 1:**
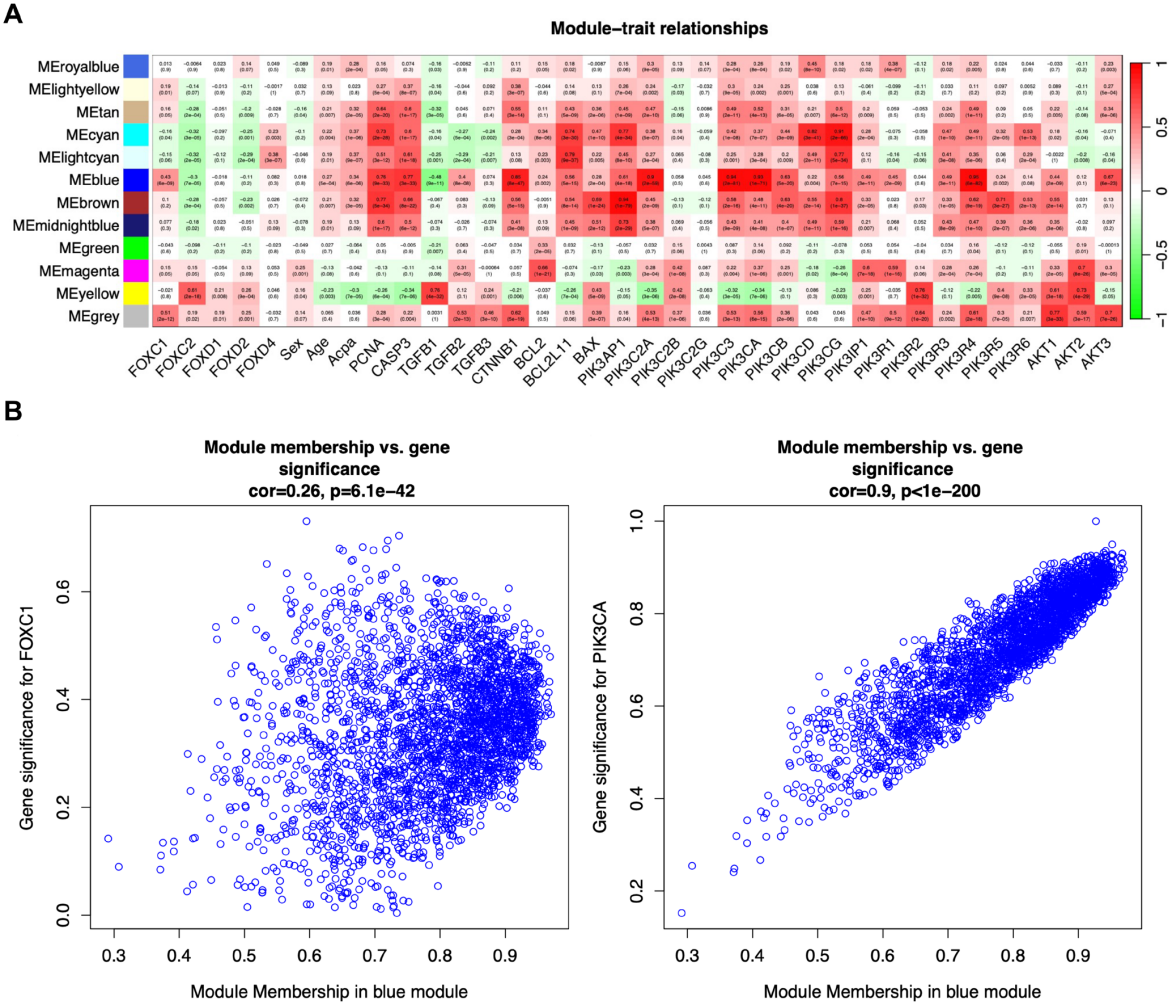
WGCNA was used to screen gene co‐expression modules and verify the functional core of the selected modules. (A) The module‐trait relationships heatmap shows the correlations between each module gene and different traits. (B) Correlation between the MM and GS for FOXC1 (left panel) and PIK3CA (right panel) with genes in the blue module.

Twelve gene co‐expression modules were obtained through WGCNA based on the optimal soft threshold, and the correlation between each module and 36 clinical traits were determined. As shown in the heatmap in Figure 1A, the correlation coefficients (cor) of FOXC1 with MEblue was 0.43 (*p* = 1 × 6^−9^) and that with MEgrey was 0.51 (*p* = 1 × 2^−12^), respectively. Likewise, PIK3CA also exhibited significant with MEblue and MEgrey were 0.93 (*p* = 1e‐71) and 0.56 (*p* = 6e‐15) (Figure 1A). To identify the modules most closely associated with the PI3K/AKT pathway, the proteins exhibiting Cor > 0.9 with the PI3K/AKT pathway proteins were screened. The blue module correlated significantly with four core proteins of the PI3K/AKT pathway, including PIK3C2A, PIK3C3, PIK3CA, and PIK3R4. We selected MEblue which was significantly associated with the target trait and analyzed the correlations between the Module Membership (MM) and the Gene Significance (GS) of the genes within this module respectively. Therefore, through WGCNA analysis, we identified the blue module as the co‐expression module most strongly associated with FOXC1 and the PI3K/AKT pathway. By analyzing the characteristic genes within this module, we aim to elucidate the intermediate mechanisms through which FOXC1 regulates the PI3K/AKT pathway. Figure 1B illustrated the internal correlations within the module. The correlation coefficient cor between the genes in this module and FOXC1 was 0.26 (*p* = 6.1e‐42), suggesting that the association between the genes in this module and FOXC1 was low‐to‐moderate. However, the correlation coefficient cor of Blue model and PIK3CA was 0.9 (*p* < 1e‐200), which was extremely strong. It is suggested that the MEblue module and PIK3CA have a highly coordinated variation. We uploaded the correlation and *p*‐values of all genes in the blue module with PIK3CA (Table S1). Although the gray module exhibits a stronger correlation with FOXC1 than the blue module (Cor = 0.51; *p* = 2e‐12), its relationship with PI3K/AKT signature proteins is less robust than that of the blue module. Therefore, we conclude that the underlying PI3K/AKT regulatory mediators are likely concealed within the blue module.

### 3.2 Screen for Overlapping Genes

**FIGURE 2:**
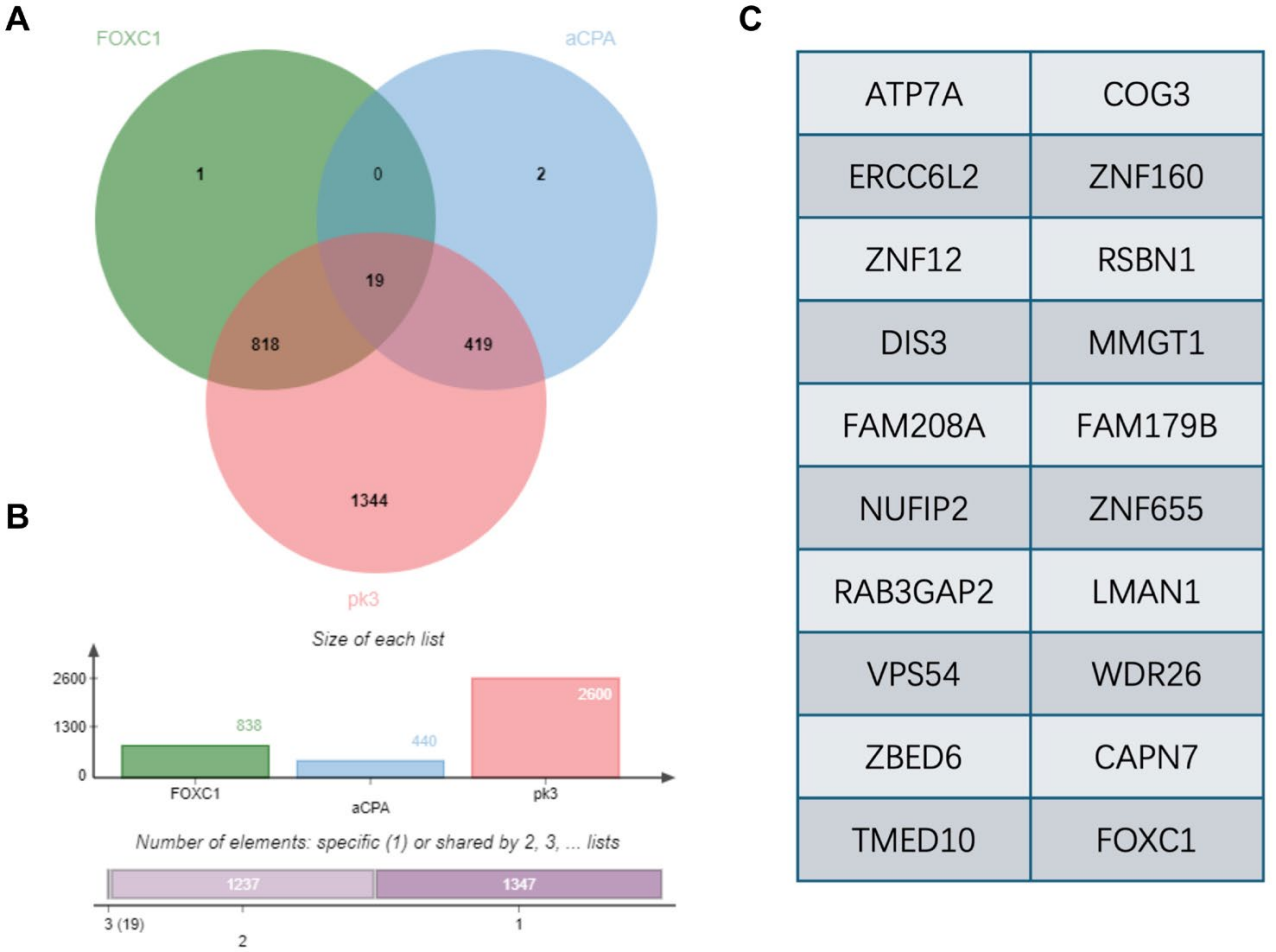
Genes co‐expressed with FOXC1, aPCA, and pk3 in RA. (A) Venn diagram shows the number of overlapping genes in the three gene lists. (B) The size of each list. (C) The numbers of genes present in one, two, and three gene lists.

The DEGs between the RA and healthy control samples were screened, and the genes co‐expressed with FOXC1, aCPA, and PK3 were identified through Venn analysis. There were 19 overlapping genes, including ATP7A, ERCC6L2, ZNF12, DIS3, FAM208A, NUFIP2, RAB3GAP2, VPS54, ZBED6, TMED10, COG3, ZNF160, RSBN1, MMGT1, FAM179B, ZNF655, LMAN1, WDR2, and CAPN (Figure 2A). As shown in Figure 2B, there were 838, 440, and 2600 genes in the FOXC1, aCPA, and PK3 lists respectively. Furthermore, 1347 genes were present only in one list, 1237 were present in two lists, and 19 were present in all three lists (Figure 2C).

### 3.3 FOXC1 Knockdown Mitigated Arthritis in the Mouse Model

**FIGURE 3:**
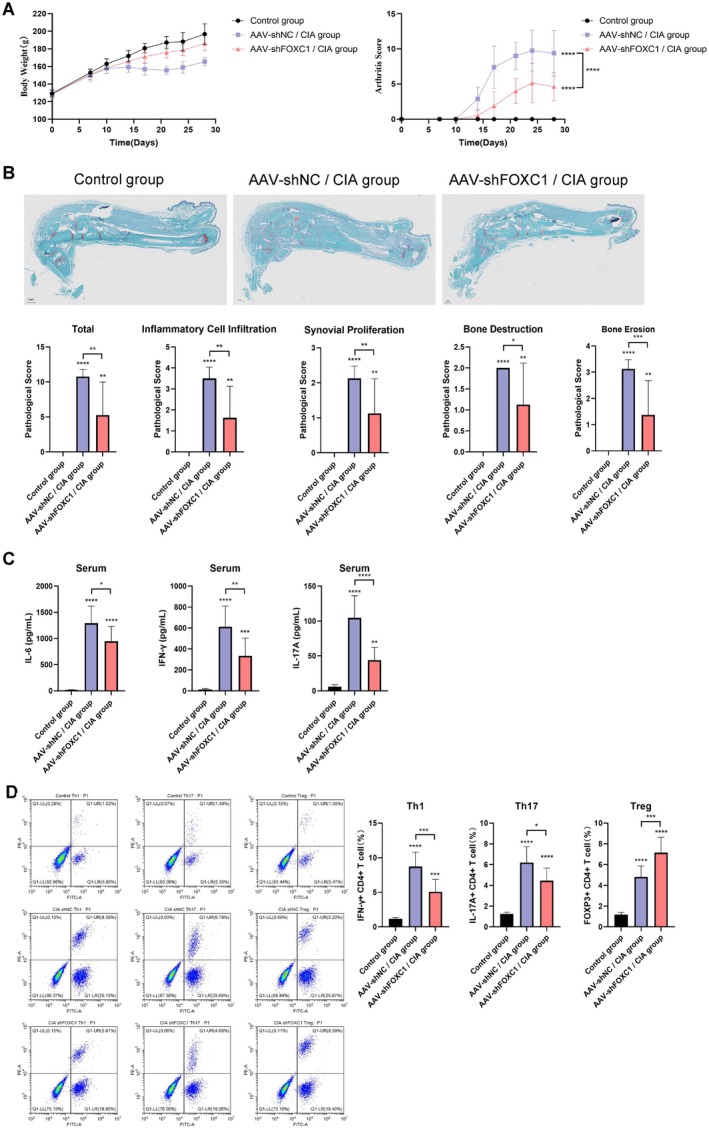
FOXC1 knockdown mitigated pathological changes in the CIA model. Two weeks after primary immunization, the CIA modeled mice were injected with AAV‐shNC or AAV‐shFOXC1. The mice were sacrificed and the splenocytes were analyzed by fluorescence‐activated cell sorting. (A) Body weight and arthritis scores of mice in the control, AAV‐shNC/CIA and AAV‐shFOXC1/CIA groups. (B) Representative images of joint sections with hematoxylin–eosin staining showing the extent of synovial proliferation, bone destruction, and inflammatory cell infiltration in the control, AAV‐shNC/CIA and AAV‐shFOXC1/CIA groups (original magnification 100×). The pathological scores of the different groups are quantified in the bar graphs (lower panel; * *p* < 0.05). (C) Serum levels of IL‐6, IFN‐γ, and IL‐17A in the indicated groups (* *p* < 0.05). (D) Flow cytometry plots and bar graphs showing the percentage of Th1 cells, Th17 cells, and Tregs in the indicated groups (* *p* < 0.05). *Note:* *p < 0.05; ** *p* < 0.01; *** *p* < 0.001; **** *p* < 0.0001.

AAV‐shFOXC1 significantly improved the severity of arthritis in the CIA modeled mice compared to that in the AAV‐shNC group (Figure 3A). Histopathological evaluation of joint sections revealed less synovial proliferation and bone destruction in the AAV‐shFOXC1‐treated CIA mice compared to the AAV‐shNC controls, although the infiltration of inflammatory cells was similar in both groups (Figure 3B). The CIA mice (AAV‐shNC control) also showed significant elevation in IL‐6, IFN‐γ, and IL‐17A levels, whereas FOXC1 knockdown reduced the secretion of these pro‐inflammatory cytokines (Figure 3C). Furthermore, the Th1 cells and Th17 cells, but not Tregs, exhibited lower abundance in AAV‐shFOXC1‐treated mice compared to the AAV‐shNC mice (Figure 3D). These findings collectively demonstrate that FOXC1 knockdown alleviates arthritis severity by suppressing synovial inflammation and remodeling the T‐cell immune response.

### 3.4 FOXC1 Promoted the Proliferation, Migration, Invasion, and Survival of MH7A Cells In Vitro

**FIGURE 4:**
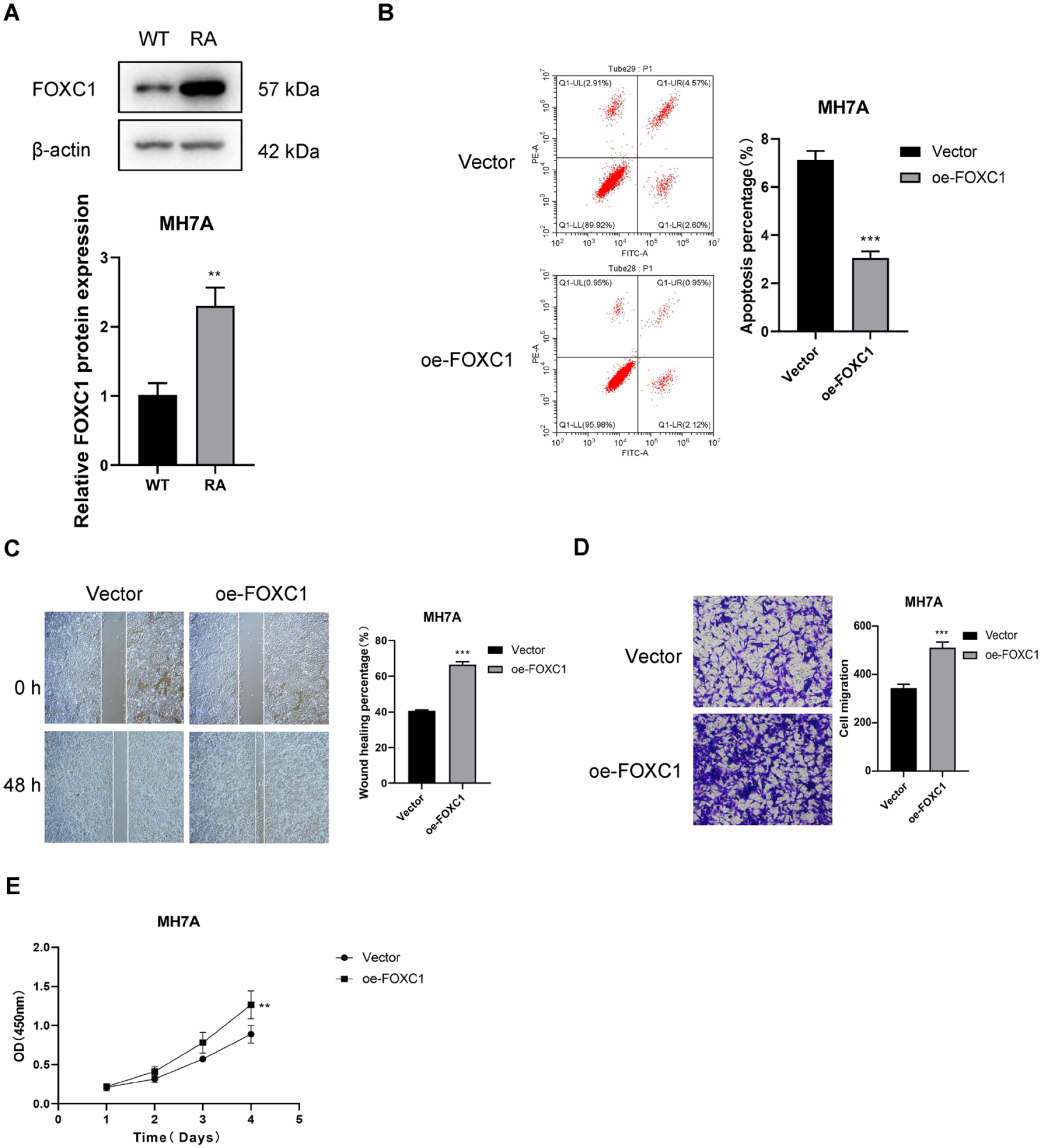
Functional verification of FOXC1 in RA model and synovial cells. (A) Immunoblot showing the differential expression of FOXC1 protein in the wild‐type (WT) and RA model. (B) Flow cytometry plots and bar graph showing apoptosis rates in the control and oe‐FOXC1 MH7A cells. (C) Representative images of scratch assay and bar graph showing the wound healing rates in the control and oe‐FOXC1 MH7A cells. (D) Representative images of Transwell assay and bar graph showing the number of invasive cells in the control and oe‐FOXC1 groups. (E) Viability rates of the control and oe‐FOXC1 MH7A cells at the indicated time points. ns *p* > 0.05; **p < 0.01; ***p < 0.001.

As shown in Figure 4A, FOXC1 protein expression was significantly higher in the synovium of the CIA model compared to that of wild‐type (WT) mice. To further explore the functional role of FOXC1 in the pathogenesis of RA, we transfected MH7A cells with FOXC1 overexpression plasmid (oe‐FOXC1) and the corresponding empty vector. The apoptosis rate in the oe‐FOXC1 group was significantly lower than that of the control group (*p* < 0.001, Figure 4B). In addition, the wound healing assay revealed higher area coverage in the oe‐FOXC1 group, suggesting that FOXC1 promotes the migration ability of RA‐FLS (*p* < 0.001, Figure 4C). Likewise, the number of invading cells in the Transwell assay was also significantly higher in the oe‐FOXC1 group compared to that in the control group, suggesting that FOXC1 promotes the invasiveness of RA‐FLS (*p* < 0.001, Figure 4D). Furthermore, the CCK‐8 assay showed that overexpression of FOXC1 increased the proliferation rate of MH7A cells in a time‐dependent manner (*p* < 0.01, Figure 4E).

### 3.5 FOXC1 Is an Upstream Regulator of ATP7A in RA‐FLS

**FIGURE 5:**
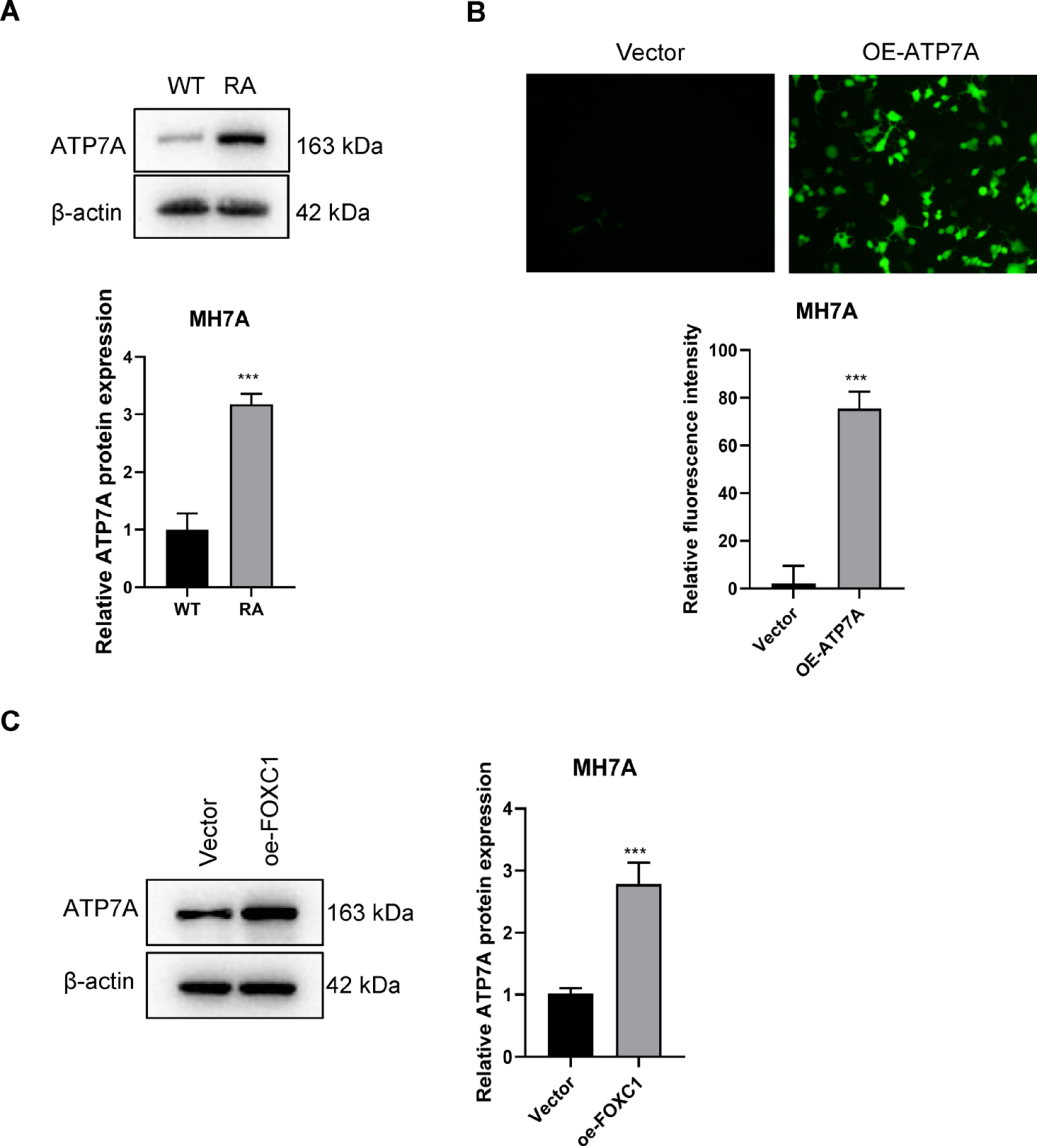
FOXC1 is the upstream regulator of ATP7A. (A) Immunoblot showing the differential expression of ATP7A protein in the wild‐type (WT) and RA model. (B) Representative images and bar graph showing the relative fluorescence intensities of Vector and OE‐ATP7A groups. (C) Immunoblot showing differential expression of ATP7A protein in the vector and oe‐FOXC1 groups. ns *p* > 0.05; ***p < 0.001.

The expression level of ATP7A protein was significantly higher in the RA model than that in WT control (*p* < 0.001, Figure 5A). In addition, ATP7A was also upregulated in the oe‐FOXC1 MH7A cells compared to that in the control cells (*p* < 0.001, Figure 5C), suggesting that FOXC1 may regulate ATP7A expression. Therefore, we next determined whether FOXC1 acts as a direct transcription factor for the ATP7A gene (see Supporting Information: Methods) through chromatin immunoprecipitation (ChIP) coupled with qPCR (ChIP‐qPCR) assay in MH7A cells. The results demonstrated a significant enrichment of the ATP7A promoter sequence in the DNA fragments precipitated by the anti‐FOXC1 antibody compared to the non‐specific IgG control, indicating that FOXC1 physically binds to the promoter region of ATP7A. To verify whether this physical binding leads to functional transcriptional activation, we constructed dual‐luciferase reporter plasmids containing either the WT or a mutated (MUT) ATP7A promoter sequence. Co‐transfection of the FOXC1 overexpression vector (pcDNA3.1‐FOXC1) with the WT‐ATP7A promoter significantly increased the relative luciferase activity by approximately 2‐fold compared to the empty vector control (*p* < 0.0001). Conversely, FOXC1‐induced enhancement of luciferase activity was completely abolished when the predicted FOXC1 binding site was mutated, and no significant difference was observed between the FOXC1‐overexpressing and control groups. Collectively, these findings provide robust evidence that FOXC1 directly binds to and transcriptionally activates the ATP7A promoter, thus identifying ATP7A as a bona fide downstream target of FOXC1 in RA‐FLS (Figure S1).

### 3.6 FOXC1 Activates the PI3K/AKT/mTOR Pathway in MH7A Cells via ATP7A

**FIGURE 6:**
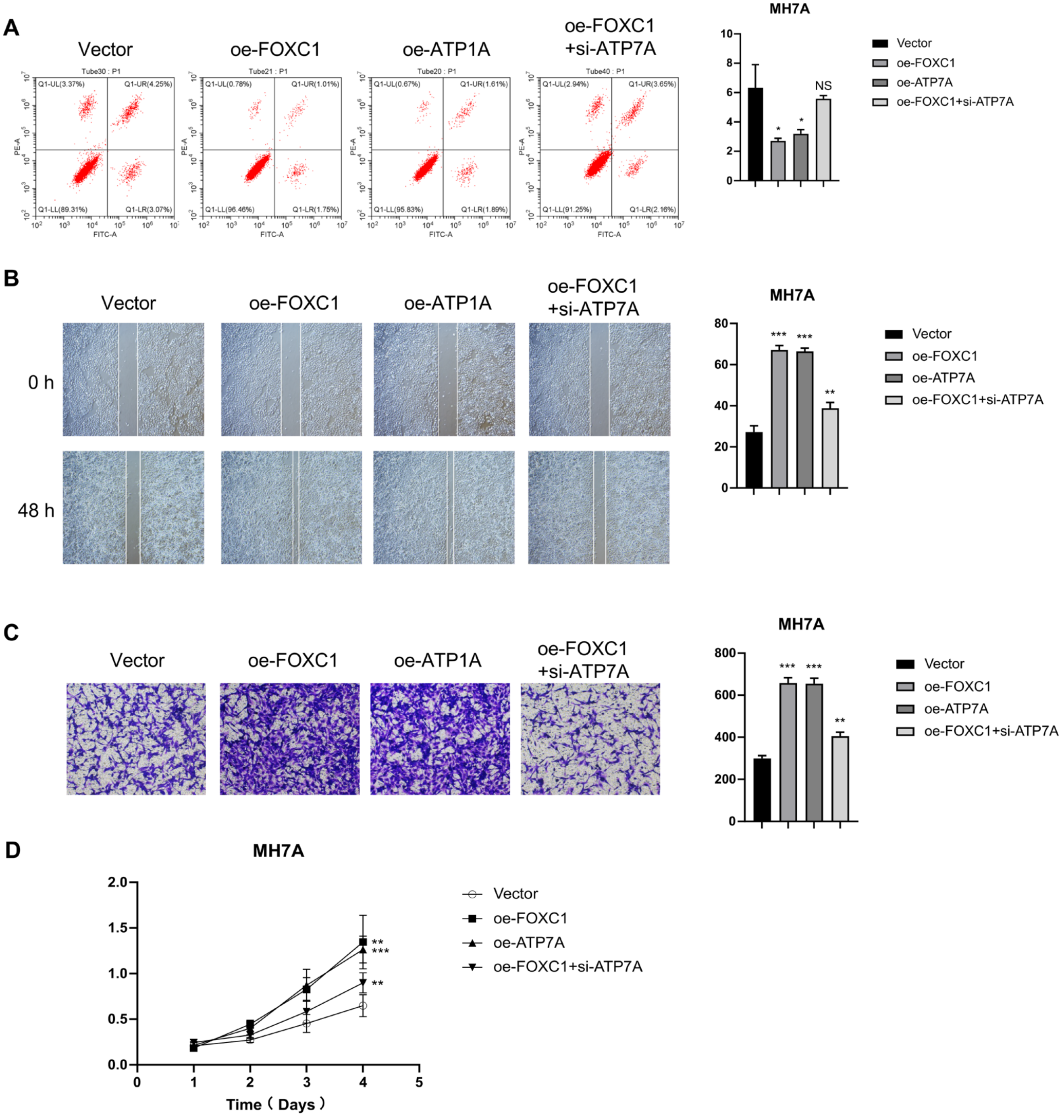
FOXC1 exerts its biological effects on MH7A cells via ATP7A. (A) Flow cytometry plots and bar graph showing apoptosis rates in the control, oe‐FOXC1, oe‐ATP7A, and oe‐FOXC1 + si‐ATP7A groups. (B) Representative images of scratch assay and bar graph showing the wound healing rates in the control, oe‐FOXC1, oe‐ATP7A, and oe‐FOXC1 + si‐ATP7A groups. (C) Representative images of Transwell assay and bar graph showing the number of invasive cells in the control, oe‐FOXC1, oe‐ATP7A, and oe‐FOXC1 + si‐ATP7A groups. (D) Viability rates in the indicated groups. ns *p* > 0.05; *p < 0.05; **p < 0.01; ***p < 0.001.

To further assess the role of ATP7A in the progression of RA, we overexpressed the gene in MH7A cells. Cells transfected with the oe‐ATP7A construct displayed intense green compared to the empty vector control group, suggesting stable overexpression of ATP7A in the MH7A cells (*p* < 0.001, Figure 5B). In addition, the oe‐FOXC1 cells were transfected with an ATP7A‐specific siRNA construct (oe‐FOXC1 + si‐ATP7A). As shown in Figure 6A, the apoptosis rates in the oe‐FOXC1 and oe‐ATP7A groups were significantly lower than that of the control (Vector) group, indicating that both FOXC1 and ATP7A protect the MH7A cells against apoptotic death. However, ATP7A silencing abrogated the effects of FOXC1 overexpression, as indicated by the higher apoptosis rates in the oe‐FOXC1 + si‐ATP7A group relative to the oe‐FOXC1 group (Figure 6A). Furthermore, the oe‐FOXC1 and oe‐ATP7A cells showed significantly higher migration rates in the wound healing assay (Figure 6B), as well as increased invasiveness in the Transwell assay (Figure 6C) compared to the control cells. However, ATP7A knockdown significantly decreased the migration and invasion capacities of the FOXC1‐overexpressing MH7A cells (Figure 6B,C). Likewise, MH7A cells overexpressing FOXC1 or ATP7A showed significantly higher proliferation rates compared to the control cells, while the growth rate of oe‐FOXC1 cells decreased with simultaneous ATP7A silencing (Figure 6D). The above results indicate that FOXC1 or ATP7A can promote the proliferation, invasion, and migration of RA‐FLS, and inhibit apoptosis, while knocking down ATP7A reverses the effects of FOXC1.

Overexpression of either FOXC1 or ATP7A significantly activated the PI3K/AKT/mTOR signaling cascade, as indicated by the marked increase in the levels of phosphorylated mTOR, PI3K, AKT, and 4E‐BP1. However, PI3K/AKT/mTOR activation in the oe‐FOXC1 group was significantly attenuated upon ATP7A knockdown (Figure S2). Notably, re‐introduction of ATP7A in the oe‐FOXC1 + si‐ATP7A cells restored the levels of p‐mTOR, p‐PI3K, p‐AKT, and p‐4E‐BP1. This “dual‐rescue” evidence strongly suggests that FOXC1 drives the activation of the PI3K/AKT/mTOR pathway in MH7A cells via ATP7A.

### 3.7 Molecular Mechanism of FOXC1 and ATP7A Action in the RA‐FLS

**FIGURE 7:**
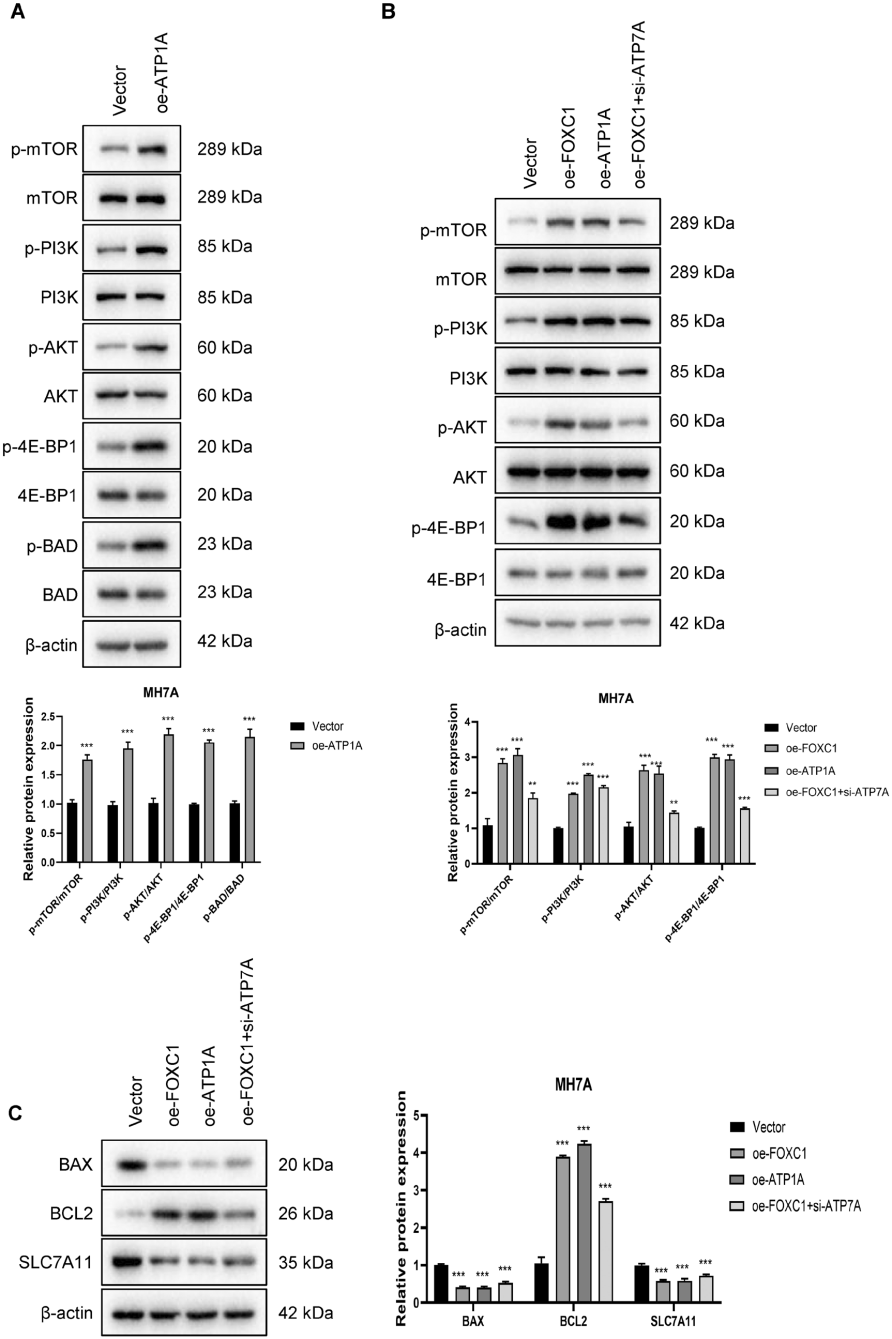
The FOXC1‐ATP7A regulates RA‐related pathways and biological processes. (A) Expression levels of proteins related to cell proliferation, survival and apoptosis in the control and oe‐ATP7A groups. (B) Expression levels of proteins related to cell proliferation, survival and apoptosis in the Vector, oe‐FOXC1, oe‐ATP7A, and oe‐FOXC1 + si‐ATP7A groups. (C) Expression levels of BAX, BCL‐2, and SLC7A11 in the indicated groups. **p < 0.01; ***p < 0.001.

To further elucidate the downstream molecular mechanisms of the FOXC1‐ATP7A axis, we investigated whether FOXC1 exerts its pathological effects through ATP7A‐dependent activation of the PI3K/AKT/mTOR pathway. As shown in Figure 7A, the expression levels of p‐mTOR, p‐PI3K, p‐AKT, p‐4E‐BP1, and p‐BAD were significantly higher in the oe‐ATP7A group compared to that in the control group. Furthermore, the above proteins were also upregulated in the oe‐FOXC1 group, while ATP7A silencing led to a partial downregulation (Figure 7B). The expression of BCL2 was significantly higher in the oe‐FOXC1 and oe‐ATP7A groups compared to the control group, while the pro‐apoptotic BAX and SLC7A11 were significantly downregulated. On the other hand, ATP7A silencing decreased BCL2 expression and increased that of BAX and SLC7A11 in the oe‐FOXC1 cells (Figure 7C). Taken together, knocking down ATP7A reversed the pathological changes induced by FOXC1 in the MH7A cells, indicating that FOXC1 exerts its biological effects through ATP7A.

To further confirm the specific effect of copper stress on cuproptosis, we assessed the expression levels of the canonical cuproptosis markers FDX1 and LIAS under copper‐stressed conditions induced by elesclomol plus Cu^2+^ (Figure S3). Treatment with the copper ionophore triggered cuproptosis in the MH7A cells, as indicated by a marked downregulation of the master regulator FDX1 and the lipoylation‐related protein LIAS. Notably, the overexpression of either FOXC1 or ATP7A significantly attenuated the copper‐induced degradation of FDX1 and LIAS proteins, indicating that the FOXC1‐ATP7A axis protects RA‐FLS from cuproptotic death by maintaining the stability of key cuproptosis‐related proteins under high copper stress. Combined with our previous findings on apoptosis and ferroptosis markers (Figure 7), these data confirm that while FOXC1‐ATP7A influences multiple cell death pathways, its ability to suppress cuproptosis through copper homeostasis is a hallmark feature of the pathogenicity of RA‐FLS.

## 4 Discussion

RA is a chronic inflammatory autoimmune disease that affects synovial joints. It can lead to cartilage and bone deformities, progressive disability, and even early death, and is frequently associated with cardiovascular, pulmonary, psychological, and skin disorders [16]. Approximately 0.5%–1% of the global population is currently affected by RA [17], and the risk is two‐ to three‐fold higher in women compared to men [18]. The etiology of RA has not been fully elucidated, although both genetic and environmental factors have been implicated [19]. For instance, smoking, obesity, exposure to ultraviolet radiation, sex hormones, and drugs can all lead to the occurrence of RA [19, 20]. Furthermore, multiple immune factors are abnormally elevated in RA patients, including autoreactive CD4 + T cells, pathogenic B cells, macrophages, inflammatory cytokines, chemokines and autoantibodies [21]. Notably, synovial macrophages release inflammatory cytokines like TNF‐α, IL‐1, and IL‐6, which stimulate the activity of FLS and exacerbate bone erosion [22]. RA patients typically present with joint tenderness, swelling, and stiffness, and may develop rheumatoid nodules, lung involvement or vasculitis, as well as systemic complications with vascular and metabolic involvement [23]. The diagnosis of RA depends on the detection of autoantibodies such as ACPA and rheumatoid factor (RF), and the treatment goals are rapid inflammation control, symptom relief, prevention of joint damage, and improvement in the quality of life [24]. Common treatment strategies include bed rest and the use of non‐steroidal anti‐inflammatory drugs and disease‐modifying anti‐rheumatic drugs (DMARDs). Despite the relatively good therapeutic effect of DMARDs, patients with RA often suffer from joint damage, disability, inability to work, and increased mortality risk within several years [25]. Therefore, RA imposes significant burden on both individuals and society, which increases exponentially with disease progression. Early diagnosis and intervention are crucial for addressing this issue, thus warranting identification of new biomarkers and therapeutic targets through in‐depth research on the underlying molecular mechanisms.

FOXC1 is a DNA‐binding transcription factor that plays a role in cellular and developmental processes, especially in the eye, bone, cardiovascular, kidney, and skin tissues [26]. It belongs to the fork head family of transcription factors and is characterized by a unique DNA‐binding fork head domain [27]. Elevated FOXC1 expression is associated with poor prognosis in various cancer types, especially basal‐like breast cancer [28]. Furthermore, FoxC1 plays a key role in the pathogenesis of RA by mediating inflammation and promoting the proliferation, migration, and invasion of synovial fibroblasts. ATP7A maintains intracellular copper homeostasis by regulating the efflux of copper ions [29]. Abnormal function of ATP7A has been implicated in Menkes disease, occipital horn syndrome (OHS), and the less common ATP7A‐related distal hereditary motor neuropathy (dHMN) [30]. However, its role in RA and the underlying mechanism remain unclear.

In this study, we screened for the DEGs in the RA patients relative to healthy controls and identified the key gene co‐expression module associated with RA. Functional annotation of the RA‐related genes revealed significant enrichment of cell proliferation and copper ion transport, as well as potential involvement in the regulation of the PI3K/AKT pathway. Furthermore, we detected high expression of FOXC1 mRNA in the synovium of RA patients, and its upregulation was correlated to genes involved in the cell cycle, DNA replication, ECM‐receptor interaction, and adhesion spots. We selected 19 genes co‐expressed with FOXC1, aCPA and PK3, and constructed a PPI network to screen for the hub genes. FOXC1 was identified as the core regulatory gene of RA. Consistent with this, the FOXC1 protein was significantly elevated in the synovium of RA patients compared to that in the healthy controls. In addition, FOXC1 overexpression in the MH7A cells significantly enhanced their proliferation, migration, and invasion while suppressing apoptosis. These characteristics define the tumor‐like aggressive phenotype of RA‐FLS [17], which is a hallmark of RA pathogenesis. Uncontrolled proliferation of FLS directly leads to the formation of the thickened synovial pannus (synovial hyperplasia), consistent with the increased synovial proliferation scores observed in the CIA mice [5]. Furthermore, the enhanced migratory and invasive capacities of these cells allow them to attach to and degrade the adjacent cartilage and bone matrix, eventually resulting in bone erosion.

Notably, ATP7A protein was upregulated in the RA patients relative to the healthy controls and also showed a significant increase in the FOXC1‐overexpressing MH7A cells. This concurred with the co‐expression of FOXC1 and ATP7A in the bioinformatics analysis and suggested transcriptional regulation of ATP7A by FOXC1. To validate this hypothesis, we established ATP7A‐overexpressing MH7A cells and knocked down ATP7A in the FOXC1‐overexpressing cells. The overexpression of FOXC1 or ATP7A markedly increased the proliferation, migration, and invasion of MH7A cells in vitro and reduced apoptosis rates. However, ATP7A knockdown reversed the effects of FOXC1 overexpression, indicating that FOXC1 exerts its biological effects—at least partly—by upregulating ATP7A. The increased efflux of copper ions due to ATP7A upregulation promotes phenotypic transformation of the cells. In addition, ATP7A‐driven reduction in intracellular copper reduces the accumulation of acylated proteins (such as DLAT) and protects the cells from copper‐induced death—also known as cuproptosis. Based on these findings, we hypothesize that the FOXC1‐ATP7A axis acts as a critical link between molecular dysregulation and the macroscopic joint damage characteristic of RA.

Given the aberrant activation of the PI3K/AKT pathway in RA, we also analyzed the impact of FOXC1 overexpression and ATP7A overexpression/silencing on the core proteins of the pathway. Overexpression of FOXC1/ATP7A significantly increased the expression levels of p‐mTOR, p‐PI3K, p‐AKT and p‐4E‐BP1, suggesting that FOXC1 and ATP7A promote the growth, proliferation, metabolism and survival of synovial fibroblasts by activating the PI3K‐AKT–mTOR signaling pathway. On the other hand, knockdown of ATP7A downregulated these proteins in the FOXC1‐overexpressing cells. Furthermore, overexpression of ATP7A significantly increased the expression of p‐BAD in the MH7A cells, suggesting that ATP7A plays a key role in apoptosis resistance. Consistent with this, FOXC1/ATP7A overexpression led to a significant upregulation of the anti‐apoptotic protein BCL‐2, and decreased the levels of the pro‐apoptotic BAX and SLC7A11. However, knocking down ATP7A in the FOXC1‐overexpressing cells significantly reduced BCL2 expression, and upregulated BAX and SLC7A11. These findings indicate that FOXC1 and ATP7A regulate apoptosis signal transduction, mitochondrial preservation, and antioxidant function, which will have to be validated experimentally. Overall, this “dual regulation” of the proliferation and survival of RA‐FLS via the FOXC1‐ATP7A‐PI3K/AKT signaling axis may amplify disease pathogenesis, resulting in persistent synovial lesions.

Although this study provides new insights for targeted RA intervention, there are certain limitations that ought to be considered. First, we did not validate the role of the FOXC1‐ATP7A‐PI3K/AKT axis in the in vivo CIA model. Our findings will have to be verified through animal experiments to clarify the physiological correlation of the mechanism. Second, the direct binding of FOXC1 to the ATP7A promoter, and the transcriptional regulation mode remain unclear and will need further elucidation. Finally, we did not systematically analyze the correlation between FOXC1, ATP7A, and the PI3K/AKT pathway in the synovial tissue of RA patients or their association with disease activity. In future studies, we will include more clinical samples to address this point.

Altogether, our study reveals a novel mechanism of RA progression involving the FOXC1‐ATP7A‐PI3K/AKT axis, which regulates the pathological phenotypes and cuproptosis of RA‐FLS. It not only deepens our understanding of the molecular pathogenesis of RA but also provides theoretical support for the development of innovative therapies targeting cuproptosis pathways, which can be combined with immunotherapies such as chimeric antigen receptor T cells [31] or DCs [32].

## 5 Conclusion

FOXC1 knockdown inhibited the proliferation, migration, and invasion of RA‐FLS in vitro and in vivo. Our study highlights the role of the FOXC1‐ATP7A‐PI3K/AKT axis in the pathogenesis of RA and provides new therapeutic targets.

## Author Contributions

D.L. and F.T. wrote the main manuscript text. Z.J. prepared Figures 1 and 2 and W.M. and prepared Figures 3, 4, 5. C.C. prepared Figure 6. L.H. support statistical analysis. Q.L. and X.Y. copyedit this manuscript. D.L. laid out this manuscript. W.L. provided funding support and an idea. All authors reviewed the manuscript.

## Funding

This research was supported by Guizhou Provincial Science and Technology Foundation Project (Natural Science) (Fundamentals of Qian Kehe‐ZK [2023] General 435, 408), Guizhou Provincial Clinical Research Center for Traditional Chinese Medicine in Rheumatic and Immune Diseases Project (Project Number: Qian Kehe Platform Talent [2020] 2202), Guizhou University of Traditional Chinese Medicine National and Provincial Science and Technology Innovation Talent Team Cultivation Project (Guizhou University of Traditional Chinese Medicine TD no. [2022]004), the Second Affiliated Hospital of Guizhou University of Traditional Chinese Medicine “Sanhang” Talent Training Project for Scientific Research Talents (SHRC‐KY2024019), and the Key Laboratory of Guizhou Provincial Education Department (Grant no. [2023]017).

## Ethics Statement

This study was conducted using cell lines and did not involve human participants, human data, or human tissue. Therefore, no ethics approval was required. However, all procedures were performed in accordance with relevant guidelines and regulations. The cell lines used in this study were obtained from public repositories or commercial sources, and all necessary permissions were obtained for their use. The authors confirm that the cell lines used are not listed in the database of commonly misidentified cell lines maintained by the International Cell Line Authentication Committee (ICLAC).

## Consent

The authors have nothing to report.

## Conflicts of Interest

The authors declare no conflicts of interest.

## Supporting information


**Figure S1:** FOXC1 directly binds to and activates the transcription of ATP7A. (A) Dual‐luciferase reporter assay in MH7A cells. Cells were co‐transfected with pcDNA3.1‐FOXC1 (or empty vector) and luciferase reporters containing the wild‐type (WT) or mutant (MUT) ATP7A promoter. Relative luciferase activity was normalized to Renilla luciferase activity (*n* = 3; ****p < 0.0001; ns, not significant). (B) ChIP‐qPCR analysis of FOXC1 binding to the ATP7A promoter. Chromatin from MH7A cells was immunoprecipitated with an anti‐FOXC1 antibody or control IgG. The enrichment of the ATP7A promoter region was quantified by qPCR. Data are presented as mean ± SEM of three independent experiments.
**Figure S2:** Genetic rescue experiments confirm the FOXC1–ATP7A–PI3K/AKT axis. (A) Western Blot and quantitative analysis of the PI3K/AKT/mTOR signaling pathway proteins in MH7A cells. The cells were divided into five groups: Vector, oe‐FOXC1, oe‐ATP7A, oe‐FOXC1 + si‐ATP7A, and oe‐FOXC1 + si‐ATP7A + oe‐ATP7A. The phosphorylation and total levels of mTOR, PI3K, AKT, and 4E‐BP1 were detected, with β‐actin as the internal control. (B) Re‐expression of ATP7A significantly rescued the signaling inhibition caused by ATP7A siRNA in FOXC1‐overexpressing cells (*n* = 3; *p < 0.05; ****p < 0.0001; ns, not significant). Data are presented as mean ± SEM.
**Figure S3:** FOXC1 and ATP7A stabilize cuproptosis‐related proteins FDX1 and LIAS. (A) Western Blot analysis and quantification of FDX1 and LIAS protein levels in MH7A cells. Cells were treated with elesclomol (50 nM) and CuCl_2_ (μM) to induce cuproptosis. The groups included: Control, Cuproptosis (elesclomol + Cu), Cuproptosis + oe‐ATP7A, and Cuproptosis + oe‐FOXC1. (B) Overexpression of the FOXC1‐ATP7A axis partially restored the protein levels of FDX1 and LIAS that were suppressed by copper stress (*n* = 3; ****p < 0.0001 versus the Cuproptosis group). Data are presented as mean ± SEM.

**Table S1:** The Raw Data of correlation and *p*‐values of all genes in the blue module with PIK3CA.

## Data Availability

The datasets generated and/or analyzed during the current study are available in the Gene Expression Omnibus (GEO) database repository at https://www.ncbi.nlm.nih.gov/geo. All data generated or analyzed during this study are included in this published article.
