# Supplementary material for: FOXC1‐ATP7A Axis Activates PI3K/AKT Signaling to Suppress Cuproptosis and Drive Fibroblast Pathogenesis in Rheumatoid Arthritis
Source: FASEB J. 2026 Apr 15;40(8):e71809. doi: 10.1096/fj.202600492R (PMC13082308; doi:10.1096/fj.202600492R)
Supplement: Supplementary file 1 — Figure S1: FOXC1 directly binds to and activates the transcription of ATP7A. (A) Dual‐luciferase reporter assay in MH7A cells. Cells were co‐transfected with pcDNA3.1‐FOXC1 (or empty vector) and luciferase reporters containing the wild‐type (WT) or mutant (MUT) ATP7A promoter. Relative luciferase activity was normalized to Renilla luciferase activity (n = 3; ****p < 0.0001; ns, not significant). (B) ChIP‐qPCR analysis of FOXC1 binding to the ATP7A promoter. Chromatin from MH7A cells was immunoprecipitated with an anti‐FOXC1 antibody or control IgG. The enrichment of the ATP7A promoter region was quantified by qPCR. Data are presented as mean ± SEM of three independent experiments. Figure S2: Genetic rescue experiments confirm the FOXC1–ATP7A–PI3K/AKT axis. (A) Western Blot and quantitative analysis of the PI3K/AKT/mTOR signaling pathway proteins in MH7A cells. The cells were divided into five groups: Vector, oe‐FOXC1, oe‐ATP7A, oe‐FOXC1 + si‐ATP7A, and oe‐FOXC1 + si‐ATP7A + oe‐ATP7A. The phosphorylation and total levels of mTOR, PI3K, AKT, and 4E‐BP1 were detected, with β‐actin as the internal control. (B) Re‐expression of ATP7A significantly rescued the signaling inhibition caused by ATP7A siRNA in FOXC1‐overexpressing cells (n = 3; *p < 0.05; ****p < 0.0001; ns, not significant). Data are presented as mean ± SEM. Figure S3: FOXC1 and ATP7A stabilize cuproptosis‐related proteins FDX1 and LIAS. (A) Western Blot analysis and quantification of FDX1 and LIAS protein levels in MH7A cells. Cells were treated with elesclomol (50 nM) and CuCl2 (μM) to induce cuproptosis. The groups included: Control, Cuproptosis (elesclomol + Cu), Cuproptosis + oe‐ATP7A, and Cuproptosis + oe‐FOXC1. (B) Overexpression of the FOXC1‐ATP7A axis partially restored the protein levels of FDX1 and LIAS that were suppressed by copper stress (n = 3; ****p < 0.0001 versus the Cuproptosis group). Data are presented as mean ± SEM. [file FSB2-40-e71809-s001.docx]

**Materials and Methods**

**1. Chromatin Immunoprecipitation (ChIP)-qPCR Assay**

The ChIP assay was performed in MH7A cells using the SimpleChIP® Enzymatic Chromatin IP Kit (Cell Signaling Technology, USA) according to the manufacturer’s instructions.

**Cross-linking and Lysis:** Approximately 1*10^7 MH7A cells were cross-linked with 1% formaldehyde at room temperature for 10 minutes, followed by quenching with 0.125 M glycine for 5 minutes. Cells were then washed with ice-cold PBS and lysed to release the nuclei. **Chromatin Fragmentation:** The chromatin was digested with Micrococcal Nuclease and further sheared by sonication to generate DNA fragments ranging from 200 to 500 bp.

**Immunoprecipitation:** The sheared chromatin was incubated overnight at 4°C with 2μg of either anti-FOXC1 antibody (Abcam, UK) or normal rabbit IgG as a negative control.

**DNA Purification and qPCR:** The protein-DNA complexes were precipitated with Protein G Magnetic Beads. After reversal of cross-links and DNA purification, the enrichment of the ATP7A promoter region was quantified by qRT-PCR using the SYBR Green method.

**Data Analysis:** The results were calculated as the percentage of input or fold enrichment relative to the IgG control.

**2. Dual-Luciferase Reporter Assay**

The transcriptional regulation of ATP7A by FOXC1 was assessed using a dual-luciferase reporter system.

**Plasmid Construction:** The human ATP7A promoter region (containing the predicted FOXC1 binding site) was amplified and cloned into the pGL3-Basic vector (Promega, USA) to generate the wild-type reporter (WT-ATP7A). The mutant reporter (MUT-ATP7A), in which the FOXC1 binding motif was altered by site-directed mutagenesis, was constructed using the QuikChange Lightning Kit (Agilent Technologies, USA).

**Cell Transfection:** MH7A cells were seeded in 24-well plates and reached 70-80% confluence before transfection. Cells were co-transfected with either pcDNA3.1-FOXC1 or pcDNA3.1-empty vector , along with WT or MUT ATP7A promoter plasmids and the pRL-TK Renilla luciferase plasmid (as an internal control) using Lipofectamine 3000.

**Luciferase Activity Measurement:** At 48 hours post-transfection, cells were harvested and lysed. Luciferase activities were measured using the Dual-Luciferase Reporter Assay System (Promega, USA) on a microplate reader.

**Calculation:** The relative luciferase activity was calculated using the following formula:

Relative Activity= Firefly Luciferase Activity/Renilla Luciferase Activity

**Supplementary Figures**


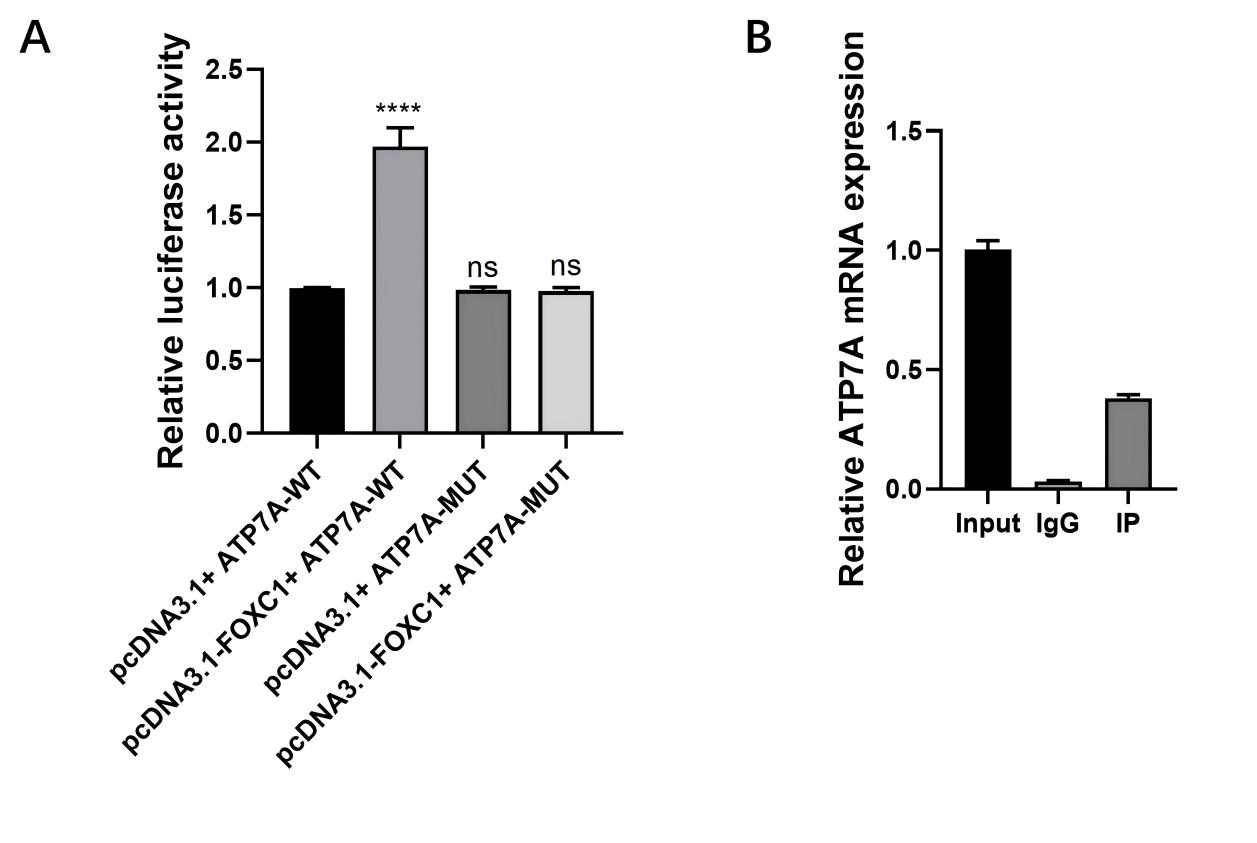


**Figure S1. FOXC1 directly binds to and activates the transcription of ATP7A.**

(A) Dual-luciferase reporter assay in MH7A cells. Cells were co-transfected with pcDNA3.1-FOXC1 (or empty vector) and luciferase reporters containing the wild-type (WT) or mutant (MUT) ATP7A promoter. Relative luciferase activity was normalized to Renilla luciferase activity (n = 3; ****P < 0.0001; ns, not significant). (B) ChIP-qPCR analysis of FOXC1 binding to the ATP7A promoter. Chromatin from MH7A cells was immunoprecipitated with an anti-FOXC1 antibody or control IgG. The enrichment of the ATP7A promoter region was quantified by qPCR. Data are presented as mean ± SEM of three independent experiments.


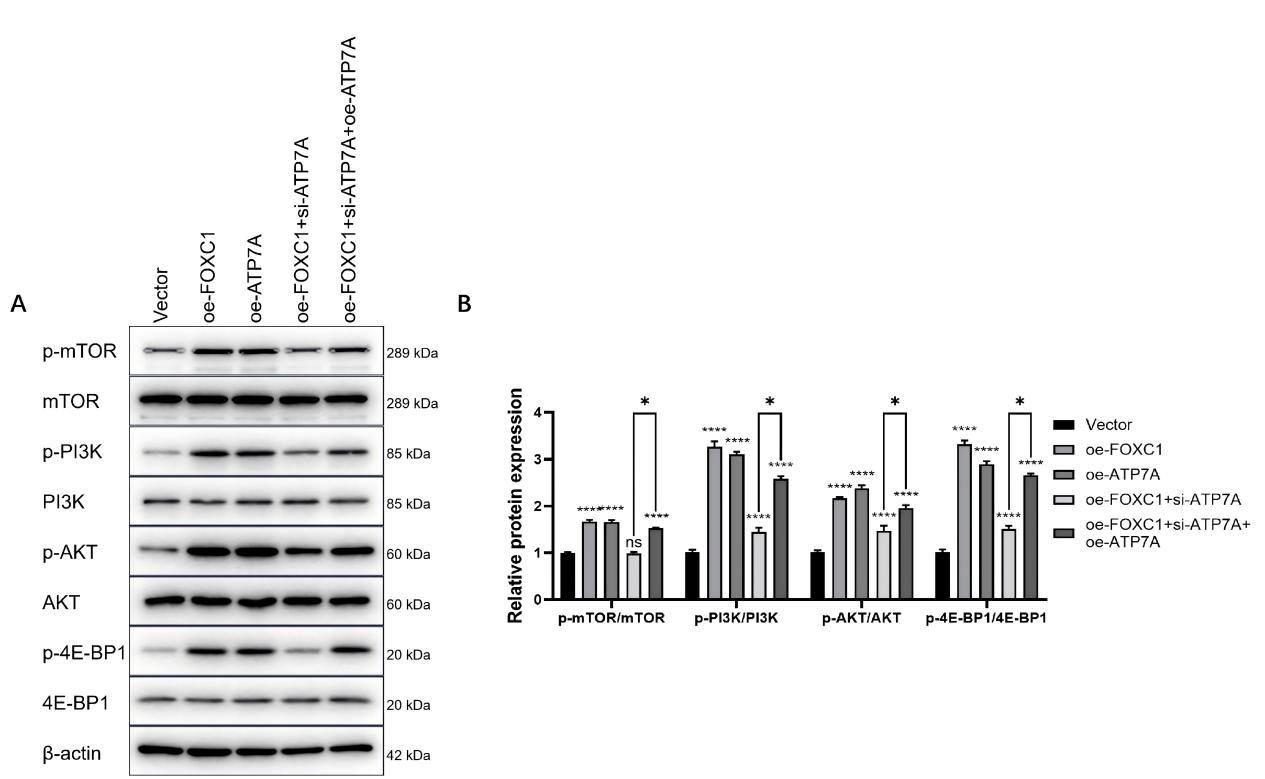


**Figure S2. Genetic rescue experiments confirm the FOXC1–ATP7A–PI3K/AKT axis.**

(A) Western Blot and quantitative analysis of the PI3K/AKT/mTOR signaling pathway proteins in MH7A cells. The cells were divided into five groups: Vector, oe-FOXC1, oe-ATP7A, oe-FOXC1 + si-ATP7A, and oe-FOXC1 + si-ATP7A + oe-ATP7A. The phosphorylation and total levels of mTOR, PI3K, AKT, and 4E-BP1 were detected, with β-actin as the internal control. (B) Re-expression of ATP7A significantly rescued the signaling inhibition caused by ATP7A siRNA in FOXC1-overexpressing cells (n = 3; *P < 0.05; ****P < 0.0001; ns, not significant). Data are presented as mean ± SEM.


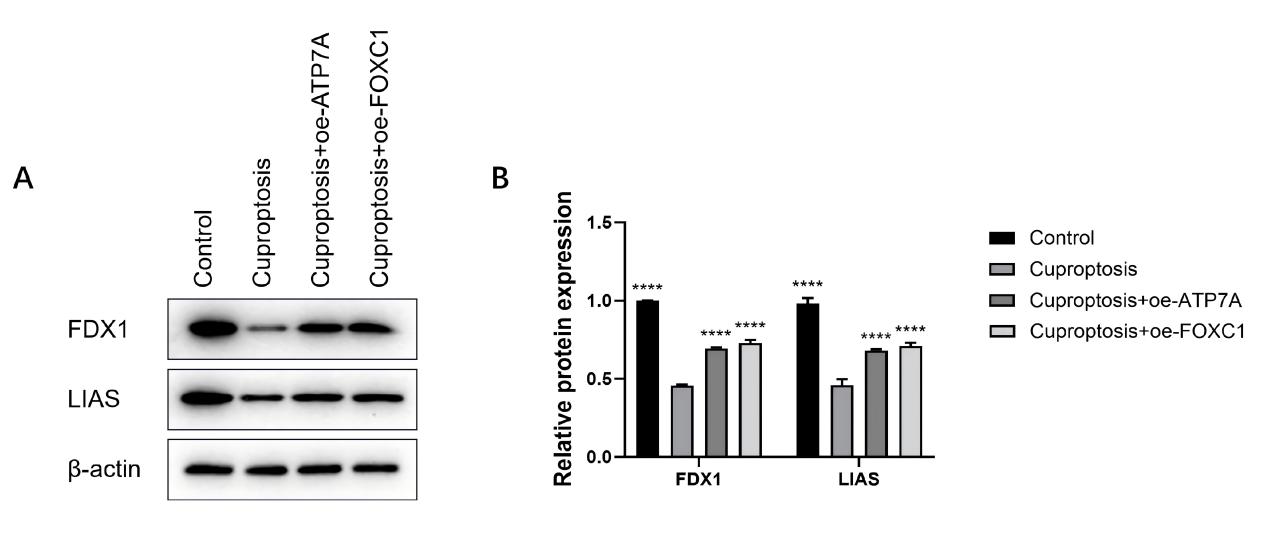


**Figure S3. FOXC1 and ATP7A stabilize cuproptosis-related proteins FDX1 and LIAS.**

(A) Western Blot analysis and quantification of FDX1 and LIAS protein levels in MH7A cells. Cells were treated with elesclomol (50 nM) and CuCl_2_ (μM) to induce cuproptosis. The groups included: Control, Cuproptosis (elesclomol + Cu), Cuproptosis + oe-ATP7A, and Cuproptosis + oe-FOXC1. (B) Overexpression of the FOXC1-ATP7A axis partially restored the protein levels of FDX1 and LIAS that were suppressed by copper stress (n = 3; ****P < 0.0001 versus the Cuproptosis group). Data are presented as mean ± SEM.
